# The Effect of Different Habitat Types and Ontogenetic Stages on the Diet Shift of a Critically Endangered Fish Species, *Coreius guichenoti* (Sauvage and Dabry de Thiersant, 1874)

**DOI:** 10.3390/ijerph15102240

**Published:** 2018-10-12

**Authors:** Zhi Yang, Xiaojuan Chen, Na Zhao, Huiyuan Tang, Jiangping Tao, Peng Zhang, Fang Shi, Chengyan Wan

**Affiliations:** 1 Key Laboratory of Ministry of Water Resources for Ecological Impacts of Hydraulic-Projects and Restoration of Aquatic Ecosystem, Institute of Hydroecology, Ministry of Water Resources & Chinese Academy of Sciences, Wuhan 430079, China; yangzhi4626@163.com (Z.Y.); chenxiaojuan@mail.ihe.ac.cn (X.C.); tanghy@mail.ihe.ac.cn (H.T.); jptao@mail.ihe.ac.cn (J.T.); fangshi.moko@gmail.com (F.S.); chywan@mail.ihe.ac.cn (C.W.); 2 State Key Laboratory of Water Resources and Hydropower Engineering Science, Wuhan University, Wuhan 430072, China; peterwin86@gmail.com

**Keywords:** diet shift, stable isotope analysis, diet plasticity, food preference, isotopic niche

## Abstract

This study examined the effect of habitat types and ontogenetic stages on the diet shift of *Coreius guichenoti* (Sauvage and Dabry de Thiersant, 1874), a critically endangered fish species. Based on the stable isotope analysis method, the following was explored: the variations in δ^13^C and δ^15^N values, isotopic niche width and four basal food sources (Mollusks, Macrocrustaceans, Aquatic insect larvae and particulate organic matters (POMs)) among three essential habitat types (the spawning ground, natural riverine feeding and nursery area, and Three Gorges Reservoir area) and between two ontogenetic stages (immature and fully mature stages). A diet shift associated with habitat type changes was observed, but there were no obvious differences in diet composition between the two ontogenetic stages. Dietary plasticity and a preference for specific foods were the important determinants of feeding behavior through the life history of this species. POM was important for the survival of this species in the resource-limited spawning ground, but this species fed more heavily on higher-order consumers in resource-abundant areas. This study highlights the importance of maintaining free connectivity among different habitats (particularly spawning grounds) to ensure the long-term sustainability of potamodromous fish species as well as the full investigation of all types of critical habitats for understanding the trophic ecology of a single fish species.

## 1. Introduction

The identification and protection of fish critical habitats are central to the active management of species at risk [1,2]. Generally, the spawning grounds and nursery, migration, and rearing areas on which fish species depend directly to complete their life history cycles, are identified as the critical habitats for fish species [1]. However, many fish species use different critical habitats within different life history stages [3,4]. The distance between critical habitats may extend to several hundred or thousands of kilometers. Due to environmental heterogeneity in different habitats, the fish species may exhibit plastic dietary patterns and diet shifts due to variation in the accessibility and availability of potential food sources [3,5,6]. Acquiring information on the diet shift among different habitats and identifying the habitat factors causing the diet shift helps guide appropriate conservation measures to protect fish resources [3].

For many fish species, diet shifts are also closely related to behavioral and morphological changes resulting from their ontogeny [7,8,9]. Generally, ontogenetic shifts in diet between size classes or life history stages are common among fish populations [7], serving as an adaptive survival strategy for resisting environmental variability. In particular, many studies have reported that the diet shift in the development stages between juvenile and adult or during early life stages, is clearly observed or estimated [8,9]. Some studies have also found that the diet shift between young individuals or young adults and adults, occurs in certain fish species [10]. However, for certain fish species, only fully mature or spawning individuals can be found in the limited spawning grounds, the diet shifts or differences between immature and fully mature stages in the spawning grounds are poorly understood, and the diet shifts of individuals between the spawning grounds and other functional habitats are also not clear. Identifying the diet shift or differences between these immature and fully mature ontogenetic stages in the spawning grounds is very important for the full understanding of the reproductive ecology of these fish species, because it provides information about the reproductive adaptation strategy to specific habitat variability [11]. Additionally, intraspecific diet competition is common for many fish species [10,12], and knowing that the intensity of intraspecific food overlaps between immature and fully mature individuals in the spawning grounds when a large number of spawning fishes aggregate in the spawning grounds is also very important for the management and conservation of fish resources. 

For characterizing diet differences among different habitats and/or ontogenetic stages, stable isotope analysis (SIA) [13,14] has been widely used in recent years. SIA has been broadly employed to track food sources over long time scales. The stable isotope ratios of carbon (δ^13^C) and nitrogen (δ^15^N) have been used most frequently. While δ^13^C has been used to trace the potential food sources of consumers, δ^15^N has been used to identify the trophic positioning of consumers, because it shows an obvious stepwise enrichment between trophic levels [14,15,16]. Based on the results of SIA, mixing models are built for estimating the proportions of source (prey) contributions to a mixture (consumer) based on biotracer data to determine diet composition, niche variation, population structure, animal movement and so on [14,17]. In recent years, Bayesian mixing models have been used most frequently, because they can improve upon simpler linear mixing models by explicitly taking into account uncertainty in source values, categorical and continuous covariates, and prior information. Depending on the R software, certain R packages (e.g., SIAR and MixSIAR), including the Bayesian mixing model, have been developed for analyzing biotracer data [17,18].

**Figure 1:**
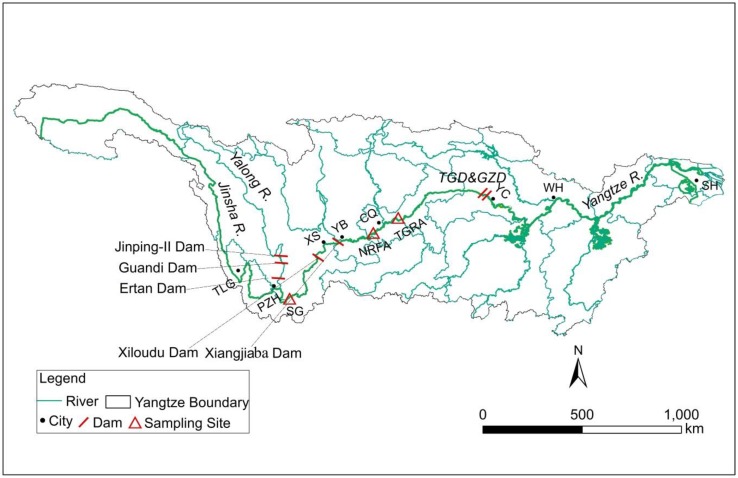
The study area and fish sampling sites in the Yangtze River Basin (SG: spawning ground; NRFA: natural riverine feeding and nursery area; TGRA: Three Gorges Reservoir area; SH: Shanghai; WH: Wuhan; YC: Yichang; CQ: Chongqing; YB: Yibin; XS: Xinshi; PZH: Panzhihua; TLG: Tiger leaping Gorge; TGD: Three Gorges Dam; GZD: Gezhou Dam).

Largemouth bronze gudgeon (LBG), *Coreius guichenoti* (Sauvage and Dabry de Thiersant, 1874), is an endemic and commercial fish species in the upper Yangtze Basin [19] that plays an important role in the transfer of energy from primary producers and consumers to top predators [20]. Due to the loss of spawning grounds and hydrologic obstructions resulting from the construction of cascaded hydropower plants on the Jinsha River (the upper segments of the upper Yangtze) beginning in 2008, this species has been listed as critically endangered on the Red List of China’s Vertebrates since 2016 [21]. The fish species spawn with drifting eggs, and their spawning grounds are only distributed in the mainstream of the middle and lower reaches of the Jinsha River as well as in the lower mainstream of the Yalong River (one of the main tributaries of the Jinsha River). Before the impoundment of the Three Gorges Reservoir (TGR) in 2003, the fish species made use of two critical natural riverine areas (the spawning area and the feeding, nursery area) during their entire life history cycles. The spawning area in the Jinsha River specifically includes the river segment from Xinshi Town to Tiger Leaping Gorges Town, while the feeding and nursery area generally consist of the river segments downstream of Xinshi Town, which can extend to the middle reaches of the Yangtze River (Figure 1). Compared to the feeding and nursery areas, the spawning areas generally display harsher natural habitat characteristics, for instance, higher mean flow velocity and sediment content, low primary productivity and fewer microhabitat types [22]. Since the impoundment of the TGR in 2003, a newly formed reservoir habitat has been added to the habitats of this fish species, possibly exerting important impacts on the population survival of the fish species [23]. However, little is known about the influences of TGR operation on the feeding ecology of this fish species.

In addition, the feeding ecology (e.g., food preference in different development stages) of LBG remains unclear, although the diet compositions of this species in certain non-spawning river sections have been documented [20,24,25]. In particular, the feeding habits of this species in the spawning grounds are poorly understood. Moreover, food preference, diet plasticity, and intraspecific competition in the spawning grounds remain unclear. In particular, the fully mature or spawning individuals of this species can only be found in the spawning grounds [19,26]; hence, whether the intraspecific competition in the spawning grounds occurs or is strengthened during the spawning season is still unknown.

In the present study, we first employ SIA to attain the values of δ^13^C and δ^15^N of LBG muscle tissues collected from three habitat types (spawning ground, natural riverine feeding and nursery area and TGR area) and then examine the differences in δ^13^C and δ^15^N among these three habitat types by use of one-way analysis of variance (ANOVA). Finally, we estimate the food preference and diet plasticity by employing MixSIAR within the R software and the intraspecific competition between immature and fully mature individuals according to the SIBE (Stable Isotope Bayesian Ellipses in R) procedure within the R package SIBR by combining the data with the collections of potential basal food sources in the three habitats. Based on the above analysis, we aim to answer the following questions: (1) Do diet shifts of LBG among three habitat types occur? (2) Does a diet shift of LBG between immature and fully mature individuals occur, and is the intraspecific competition between immature and fully mature individuals obvious in the spawning ground during the spawning season? (3) What are the main food sources of LBG in different habitats, and does the species display evident diet plasticity? (4) What effect has the formation of the TGR had on this species? 

## 2. Materials and Methods

### 2.1. Study Area

The study was carried out at three sites in the Yangtze River basin representing the three habitat types of LBG (Figure 1). The spawning ground sampling site (SG) located in Jiaopingdu (Kunming, Yunnan Province) is a natural riverine segment, where the substrates are mainly composed of boulders and large gravels [27]. Due to the rapid velocity (generally >2 m/s) and high sediment concentration in the water columns year-round, this river segment generally exhibits low biomasses of plankton and zoobenthos. The natural riverine feeding and nursery area sampling site (NRFA) located in the Jiangjin site (southwestern Chongqing Municipality) is also a natural riverine segment with a wide river surface (generally >200 m in river width), exhibiting the complex flow regime and the diversified substrate combinations that are beneficial as foraging and spawning grounds for many fish species [28]. The TGR area sampling site (TGRA) located in the Fuling District (central Chongqing Municipality) is the newly formed reservoir section where the bed substrates are mainly dominated by small-diameter sands and muds and display low water flow velocity and enriched nutrient loading [23]. Before the sampling period, two vast hydropower reservoirs (the Xiangjiaba and Xiluodu Reservoirs) were filled in October 2012 and May 2013, respectively. During the filling periods of the above two reservoirs, important potamodromous fish species (including the species LBG) were captured by gillnets and hooks from the sections downstream of the Xiangjiaba Dam and then transported to the upstream of the Xiluodu Reservoir. However, no fish passage facilities were constructed on these two dams, although a feasibility analysis of fish passage construction involving these two dams had been extensively discussed. 

### 2.2. Sample Collection and Processing

Fish sample collections in the SG, NRFA and TGRA were taken from 5 June to 16 June 2013. Care of animals used in scientific procedures was under supervision of the Ethics Committee of the Institute of Hydroecology, Ministry of Water Resources and Chinese Academy of Sciences. The survey period was during the main spawning season of LBG that produced obvious peaks in egg and larvae numbers [19]. Fish specimens were collected using gillnets with mesh sizes of 1.5, 3, 5, 5.5, 8 and 10 cm. The gillnets were usually placed in the water column from dusk until dawn depending on the fishermen’s experience. Generally, a total of 6–12 gillnets were used on each sampling day at each sampling site. For the SG site, due to the construction of cascade dams (three dams have been built downstream of the spawning grounds of LBG) in the lower reaches of the Yalong River since 2000, the LBG individuals upstream of the Jinping-II Hydropower Station (Figure 1) had difficultly passing through the three dams to the SG site [26]; hence, the fish specimens in this study area were mainly collected from the Jinsha River population. A total of 175 specimens were collected, including 68 individuals from SG, 55 from NRFA, and 52 from TGRA. All individuals were collected and measured (total length, TL; standard length, SL, measured to the nearest 1 cm) and weighed (body weight, BW, measured to the nearest 0.1 g). Simultaneously, through dissection, the gonad development of LBG was classified as six stages: (I) immature fish stage; (II) quiescent stage; (III) ripening stage; (IV) ripeness stage; (V) reproduction stage; (VI) spent stage by visual observation [29]. In this study, we defined the individuals in the IV–VI stages as fully mature individuals and the other stages as immature individuals [29]. In addition, scales from all collected fish were extracted from the area below the dorsal fin and above the lateral line as the age-determining materials [30]. The scales were first cleaned with water and NaOH, placed between two slides and then observed in the field using a SZ61TR stereo microscope (Olympus, Japan) to determine the age of each fish [30]. After removing the skins and scales, muscle tissues were taken from below the dorsal fin. These tissues were frozen and freeze-dried for 72 h and stored in glass vials at −20 °C until further analysis [31].

At the same time as the fish sampling, the water temperature (WT, °C), dissolved oxygen (DO, mg/L), pH, and velocity of water flow (m/s) were measured in situ using YSI 6600 (YSI Inc., Yellow Springs, OH, USA) and LGY II (Nanjing Shengrong Instrument and Equipment Inc., Nanjing, China) instruments. Samples were simultaneously used to determine chemical parameters, including total nitrogen (TN, mg/L), total phosphorus (TP, mg/L), biochemical oxygen demand (BOD_5_, mg/L), and chemical oxygen demand (COD_Mn_, mg/L), according to standard methods (Environmental Quality Standards for Surface Water, GB3838-2002) (SEPA and AQSIQ, 2002). Based on the modified criteria of Jiang et al. [32], the predominant substrate was assigned to one of three types: (1) cobble plus boulder (CPB), (2) pebble plus gravel (PPG), or (3) sand plus silt (SPS). 

According to previous gut-content data [24,25], the four primary food source types (mollusks, macrocrustaceans, aquatic insect larvae and particulate organic matter (POM)) for LGB were collected at the three sampling sites during the fish sample collection. POM was collected with a phytoplankton net, and then samples were filtered using a filtration apparatus coupled to a vacuum pump and previously calcined filters (Whatman QM-A quartz-fiber filters) [33]. Macrocrustacean samples (crabs and shrimps, including *Macrobrachium nipponense*, *Procambarus clarki*, *Exopalaemon modestus*, *Caridina* and *Sinopotamon*) were collected using 1.5 cm mesh nylon trap nets. Mollusks (snails and *Limnoperna fortunei*) were collected using Peterson samplers in areas of slack water or directly by hand from submerged rocks in the sampling section [20]. Mollusk specimens were placed in aerated distilled water overnight to eliminate intestinal contents, and their shells and soft tissue were then collected [20]. Aquatic insect larvae, mainly composed of species of Trichoptera, Odonata and Ephemerida, were captured from shoal areas using hand nets [20]. A total of 7 mollusks, 11 macrocrustaceans, 10 aquatic insect larvae and 6 POM samples were collected in SG. A total of 10 mollusks, 9 crustaceans, 11 aquatic insect larvae and 6 POM samples were collected in NRFA. In total, 8 mollusks, 12 crustaceans, 12 aquatic insect larvae and 6 POM samples were collected in TGRA. All collected samples of potential food sources were frozen at −20 °C in the field, sealed in Styrofoam containers and transported to the laboratory for further processing.

All samples were dried at 60 °C for 24 h in an air-circulated oven, after which lipids were removed by extracting the samples in chloroform-methanol 2:1 by volume overnight at room temperature [34]. These samples were then independently homogenized using a mortar and pestle and then weighed to the appropriate mass for SIA [31]. Approximately 2 mg powder was weighed and encapsulated for animal samples, whereas 15–30 mg was taken for POM. The δ^13^C and δ^15^N were measured using an IsoPrime100 stable isotope ratio mass spectrometer equipped with a vario PYRO cube elemental analyzer (Environmental Stable Isotope Lab., Chinese Academy of Agricultural Sciences, Beijing) and expressed as parts per thousand (‰) relative to standards. The Vienna Pee Dee Belemnite and atmospheric N_2_ were used as the standards for carbon and nitrogen, respectively [33].

### 2.3. Data Analysis

One-way analysis of variance (ANOVA) was first used to determine whether δ^13^C and δ^15^N values varied significantly among the three habitat types [35]. Prior to the ANOVA, the Kolmogorov–Smirnov test and the Bartlett test were conducted. To meet these two test requirements, the parameters (δ^13^C and δ^15^N values) were transformed by dividing by the respective minimum value. If significance was detected, the Tukey multiple comparison test was conducted, or a nonparametric Kruskal–Wallis test was conducted. Following the gonadal examination, the samples in the SG were classified as two ontogenetic stages, including the fully matured stage (FMS) and the immature stage (IS). The independent-samples *t* test was chosen to identify the differences in δ^13^C and δ^15^N values between FMS and IS, revealing the diet differences between immature individuals and fully mature individuals in the SG. 

The SIBER procedure (Stable isotope Bayesian Ellipses in R) [36] within the R package SIAR [37] was applied for examining variation in the trophic niche among the three habitat types (SG, NRFA and TGRA) and between the two ontogenetic stages (FMS and IS). The SIBER procedure is based on the concept that multiple stable isotope ratios measured from consumers represent niche dimensions, and broadly varying stable isotope ratios are indicative of a wider consumer isotopic niche. Here, we used Bayesian Standard Area Ellipses (SEA_B_, also called isotopic niches area) for comparing individual groups with each other and the Monte Carlo Markov-Chain simulation for constructing ellipses characterizing isotopic variation that provide a robust indicator of isotope niche width [14]. Simultaneously, by calculating the probabilities from posterior distributions of the parameters of model given the prior data, the differences in SEA_B_ between different groups were estimated. To conduct these comparisons, the maximum-likelihood-based probabilities were calculated [14].

Finally, the Bayesian mixing model MixSIAR [38] was used to determine the likely contribution of potential food sources to the diets of LBG in three habitat types (SG, NRFA and TGRA) and in two ontogenetic stages (FMS and IS). The MixSIAR model is written in the open-source language R. In this model, the values of two biotracers (δ^13^C and δ^15^N) of the 175 collected LBG individuals were used as the mixture data, the mean values and standard deviations of δ^13^C and δ^15^N in the four potential foods as the food source data, and the discrimination data were acquired by referring to published literature that involved the study areas of this paper [20,39]. The habitat type or the ontogenetic stage in the spawning ground in this model was considered as the fixed effect, because the diet of each group was separately estimated, rather than the overall diet [38]. Prior to model analysis, we first plotted consumer and source isotope data to ensure that the source data bound the consumer data and then used the diagnostic tests (Gelmin-Rubin and Geweke) and trace plots to examine the model convergence [38]. Finally, the proportional contributions of each potential food estimated from the mixing models were reported as range estimates rather than limited to the mean to avoid misrepresenting the uniqueness of the results [40]. 

All statistical analyses were performed using R software [41], and significance levels for all analyses were set to *p* < 0.05.

## 3. Results

### 3.1. Characteristics of Specimens and Sampling Sites

A total of 175 LBG samples were collected from the three sampling sites (SG, NRFA and TGRA). The mean values and distribution ranges of total length, standard length, body weight and age for immature and fully mature individuals in the three sampling sites are shown in Table 1. For the immature individuals, the mean values of total length, standard length, body weight and age in NRTA were greater than those in SG and in TGRA.

For three sampling sites, eight physical and chemical parameters showed significant differences among the three habitat types (SG, NRFA and TGRA, Table 2). The velocity of water flow and CPB generally decreased from the SG to the TGRA. Conversely, the physiochemical parameters, including pH and SPS, showed significantly increasing patterns from SG or NRFA to TGRA (Table 2).

### 3.2. Comparisons of Carbon and Nitrogen Stable Isotope Ratios among the Three Habitat Types (SG, NRFA and TGRA) and between Two Ontogenetic Stages (FMS and IS)

**Figure 2:**
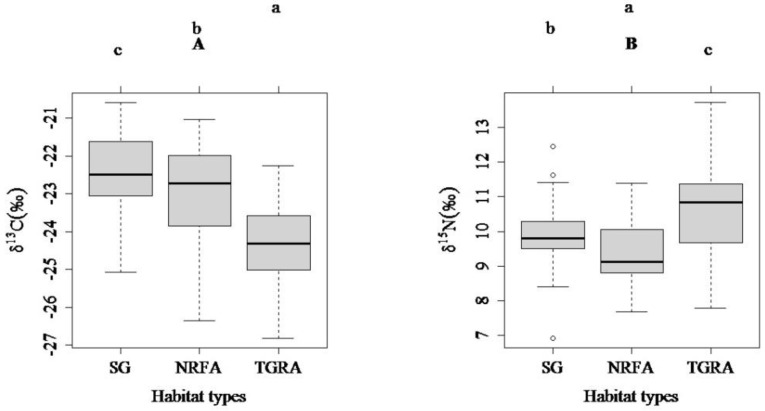
Comparisons of δ^13^C (**A**) and δ^15^N (**B**) values among three habitat types using Tukey’s test (different lowercase letters indicate significant differences; SG: spawning ground; NRFA: the natural riverine feeding and nursery area; TGRA: The Three Gorges Reservoir area; boxes show interquartile ranges, the horizontal bar indicates the median value, and whiskers reflect 1.5× the interquartile range).

Comparisons of δ^13^C and δ^15^N values across this study period (5 June to 16 June 2013) using one-way ANOVA showed strong evidence of isotopic differences among the three habitat types (Figure 2; δ^13^C: *F* = 50.88, *p* < 0.001; δ^15^N: *F* = 21.80, *p* < 0.001). The mean δ^13^C values at SG were significantly greater than those at NRFA and TGRA (*p* = 0.007 and *p* < 0.001), while the mean δ^15^N values at NRFA were lower than those at SG and TGRA (both *p_s_* < 0.001) (Figure 2).

**Figure 3:**
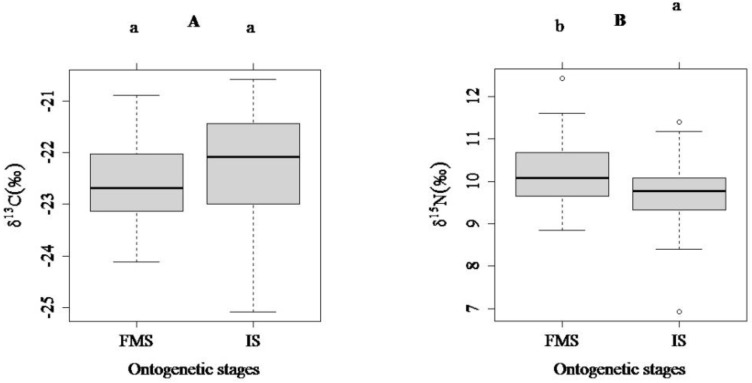
Comparisons for δ^13^C(**A**) and δ^15^N (**B**) values between two ontogenetic stages (different lowercase letters indicate significant differences; SG: spawning ground; NRFA: the natural riverine feeding and nursery area; TGRA: The Three Gorges Reservoir area; FMS, fully mature stage; IS, immature stage; boxes show interquartile ranges, the horizontal bar indicates the median value, and whiskers reflect 1.5× the interquartile range).

No significant difference was detected for δ^13^C values between the two ontogenetic stages, while strong evidence of δ^15^N difference between the two ontogenetic stages was detected (Figure 3; *t* test, δ^13^C: *t* = 1.50, *p* = 0.14; δ^15^N: *t* = −2.52, *p* < 0.001).

### 3.3. Variations in Isotopic Niche Widths among Three Habitat Types (SG, NRFA and TGRA) and between Two Ontogenetic Stages (FMS and IS)

**Figure 4:**
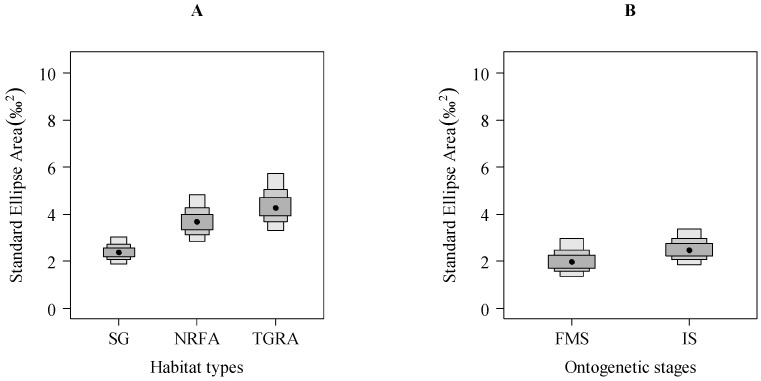
Variation in isotopic width (SEA_B_) among three habitat types (**A**) and between two ontogenetic stages (**B**) (SG: spawning ground; NRFA: the natural riverine feeding and nursery area; TGRA: The Three Gorges Reservoir area; FMS, fully mature stage; IS, immature stage; boxes represent the 50, 75 and 95% Bayesian credibility intervals).

The standard area ellipses (SEA_B_) among habitat types and between ontogenetic stages are shown in Figure 4. Compared to the individuals from SG or NRFA, the individuals from TGRA had the largest mean (95% credibility limits) isotopic niche width of 4.41 (3.30–5.73)‰^2^ (Figure 4A). However, the maximum-likelihood pairwise comparison showed that there were obvious differences between the individuals from SG and from NRFA (probability (*p*) = 99.27%) and between the individuals from SG and from TGRA (*p* = 99.95%), whereas there were no significant differences (*p* = 80.10%) between the individuals from NRFA and from TGRA. The mean SEA_B_ in FMS individuals (2.12‰^2^) was lower than that in IS individuals (2.58‰^2^) (Figure 4B), but there was only an 80.20% probability of differences in isotopic niche width between these two ontogenetic stages. 

### 3.4. Differences in Potential Food Sources among the Three Habitat Types (SG, NRFA and TGRA) and between Two Ontogenetic Stages (FMS and IS)

For all four potential food sources, significant differences in δ^13^C and δ^15^N were observed among the three sampling sites (all *P_s_* < 0.05, Table 3). From SG to TGRA, an increasing δ^13^C gradient in aquatic insect larvae and POM was observed, while δ^15^N values for these two prey items showed declining trends (Table 3). For mollusks, the δ^13^C and δ^15^N values displayed a decreasing trend from SG to NRFA or TGRA, but for macrocrustaceans, the δ^13^C values showed an increasing (from SG to NRFA) and decreasing (from NRFA to TGRA) trend, while the δ^15^N values displayed a decreasing gradient from SG to TGRA (Table 3).

The percentages of potential food contributions for LBG among the different habitats were determined using MixSIAR and are displayed in Table 4. The contributions of potential food sources to LBG showed higher proportions of POM and lower proportions of aquatic insect larvae, macrocrustaceans and mollusks in SG than in NRFA and TGRA. POM was identified as the main food sources for LBG in SG, and its mean contribution decreased from 91.0% in SG to 34.4% in NRFA and then to 1.4% in TGRA. In NRFA, the POM and macrocrustaceans became the main food sources for LBG (34.4%; 8.9–73.9% credible interval (CI) and 56.4%; 16.8–82.8% CI, respectively). In TGRA, the main food sources for LBG were mollusks and macrocrustaceans (47.4%; 31.9–61.4% CI and 49.4%; 35.6–65.0% CI, respectively).

For the FMS and IS individuals, POM contributed the highest proportion for LBG (85.1%; 74.4–93.7% CI and 94.2%; 84.3–98.9%, respectively), and the other three potential food sources displayed little contribution (Table 5). However, the contributions of aquatic insect larvae, macrocrustaceans and mollusks to FMS were greater than the contributions of these three food sources to IS (Table 5).

## 4. Discussion

Previous studies found that differences in diet were strongly related to variations in physical and chemical parameters at different ecological scales [3,33]. In the present study, significant variation existed in the isotope signatures of LBG muscle tissues among three highly heterogeneous habitats, indicating an obvious diet shift among three habitats (SG, NRFA and TGRA). This finding indicated that habitat attributes played an important role in the diet composition of LBG. In particular, the diet shift between NRFA and TGRA showed that the newly-formed reservoir can obviously influence the diet composition of LBG. In addition, the diet shifts among the three habitat types may highlight the importance of maintaining habitat connectivity between different habitat types when considering the completeness of the life cycle. Due to the high velocity of water flow (generally >2 m/s, Table 2), the SG displayed low primary productivity and only provided limited foods for LBG, while the NRFA and TGRA showed relatively low water flow velocity and enriched nutrient levels [23], which can support the survival and maturation of more individuals. The movement between habitats may be a life history strategy for long-term adaptation to environmental variation. 

Many studies have also documented that diet shift for a given fish was closely associated with maturation and spawning [42,43]. Fuiman and Faulk [44] even found that the diet shift in a marine batch-spawning fish could impact the transfer of arachidonic acid from diet to egg. However, in the present study, the results provided no statistical support for the difference in δ^13^C values between immature and fully mature stages in SG, but a significant difference in δ^15^N values between these two stages was detected. These results might imply that for LBG, the diet shift or the difference between immature and fully mature stages was not obvious even though the food intake of these two developmental stages was different. In addition, the individuals investigated in this study only included the young, subadult and adult individuals but did not include larvae or juvenile stages because of the lack of distribution of these life stages in spawning grounds. Gao et al. [45] reported that a large number of larvae and juveniles of LBG could be collected only downstream of Xinshi Town (Figure 1). 

Previous studies have used muscle tissue to study the isotopic shift between ontogenetic stages (particularly during early ontogeny) [7,8]. In the present study, we used muscle tissue to compare the isotopic difference between immature and fully mature stages based upon a complete investigation of the life history characters of LBG. LBG is a long-lived fish species, and its growth inflexion age is 8.13 years [46], indicating that fish individuals collected in this study (Table 1, age range 3–6 years) still exhibited a rapid growth speed. Thus, sufficient nutrients were available to these individuals to meet growth needs. Additionally, LBG produces eggs and spawns many times in its life [26]; therefore, this species must allocate enough nutrients to muscle tissue in order to adapt to the harsh conditions (e.g., high stream velocity and low diet biomass) of the spawning grounds. 

Many migratory fishes typically move or migrate to the spawning grounds with limited areas for spawning [47,48], resulting in an obvious increase in population density in spawning grounds during the spawning seasons. This behavior likely intensifies the competition among individuals for foods. In particular, for certain fish that spawn in the upper segments of one large river (with limited food availability), their spawning aggregations may lead to the more serious food shortages to the presence of local populations of the same fish species [49]. Our study showed no significant differences in isotopic niche width between immature and fully mature individuals in SG, indicating the same prey sources for individuals of these two ontogenetic stages. Obviously, marked intraspecific food competition and similar feeding ecology existed between these two ontogenetic stages. Additionally, this finding suggested relatively limited aggregation times of LBG reproductive cohorts in the spawning ground, because of the very similar ecological niche between immature and fully mature individuals and limited food availability in the spawning ground. Fish aggregate when spawning and leave after spawning. Tao et al. [50] reported that LBG aggregated into the spawning grounds for spawning during the flood and departed after spawning. In this context, maintaining the undisturbed state of LBG spawning grounds during the spawning season is vital for the protection of this fish species. 

There is a longstanding argument about the diet preference of LBG. Huang and Deng [24] and Liu et al. [25] found that LBG was an omnivorous fish with carnivorous preference by using stomach content analysis (SCA) based on the samples from two feeding grounds of LBG, while Li et al. [20] used stable isotope analysis (SIA) and found that LBG was an omnivorous fish with phytophagous preference based upon the samples from another feeding ground of LBG. Our results confirmed that LBG was an omnivorous fish with a carnivorous preference based upon the samples collected across all critical habitat types. Our results were consistent with the SCA results [24,25], but were obviously different from the SIA results [20]. This difference in SIA results possibly resulted from the differences in sample processing and statistical methods. Li et al. [20] did not remove lipids in sample processing and used the IsoSource software for determining the likely contribution of potential food sources to the diets of LBG, both of which likely produced obvious deviations from the true values. Post et al. [51] noted that the lipids in samples should be removed when the C/N ratio of samples is >4 because a high lipid content possibly affects the δ^13^C value. Moore and Semmens [17] determined that although the IsoSource model can address the uncertainty associated with more sources than an analytical solution, it does not formally incorporate the variation in isotope signatures or fractionation. When used to analyze the same data, IsoSource generally obtained different results from the MixSIAR model [17]. Many studies have suggested that a combination of SCA and SIA is more suitable than using SCA alone to determine temporal and regional variations in the diet compositions of certain fish species [52,53]. However, in view of the uneven digestion and assimilation rates of different potential foods [53], we chose the SIA for determining the potential food sources of LBG. In addition, our findings demonstrated that the determination of diet for a fish species, especially the food preference of an omnivorous species, must include analysis across all critical habitats.

Our MixSIAR output indicated that POM was the dominant food source for LBG in the spawning ground (SG), while macrocrustaceans and POM were the major diet components for LBG in the feeding and nursery ground (NRFA). In particular, macrocrustaceans constituted the highest proportion of the LBG diet in NRFA. This finding implied that diet plasticity occurred from the spawning ground to the feeding and nursery ground. This fish species can change its feeding behavior by adapting to the food species composition in the surrounding environment. The main food sources of LBG became macrocrustaceans and mollusks when LBG entered the TGR, further confirming the dietary plasticity of LBG. Furthermore, the isotopic niche widths became wider from the spawning ground to the river section of the reservoir area. These results indicate that downstream feeding grounds, especially the reservoir area, can provide more diversified and abundant food sources. This variation pattern seems common in many fish populations of dietary specialists [54,55], which is possibly an adaptation mechanism for decreasing mortality at the early life history stage. LBG spawn pelagic eggs in resource-limited areas, but its larvae and juveniles grow up in areas with abundant food [19]. This condition is likely a typical case for obtaining the optimal habitat suitability by diet plasticity.

Compared to immature individuals, the fully mature individuals feed on higher proportions of aquatic insect larvae, macrocrustaceans and mollusks, although the difference between these two groups of individuals was not significant. This possibly resulted from the size-related difference [7]. In this study, the body sizes of fully mature individuals were obviously larger than those of immature individuals, implying that fully mature individuals likely exhibit a greater ability to feed on larger sizes of macrocrustaceans and mollusks. However, the POM still played a very important role in the spawning ground, suggesting the importance of the inputs of terrestrial organic detritus into the upper segments of the river [56] because these river segments generally displayed low concentrations of autochthonous algae and endogenous organic detritus [57].

In addition, the isotope niche width of LBG individuals in the reservoir area was broader than that of both other sampling sites. The results showed that the individuals in the reservoir area fed more heavily on higher-order consumers, suggesting an important function of the newly formed reservoir area as the feeding grounds of LBG. However, there are seventeen very large dams in operation, under construction, or planned, on the mainstem of the upper Yangtze River [57]. This activity will inevitably block the migratory routes of LBG from the feeding and nursery grounds to the spawning grounds, inducing a partial or even total loss of the spawning grounds upstream of Xiludu Dam [57]. Although certain sections of TGR (in particular of the tail of the reservoir) can serve as the feeding grounds of LBG, they cannot function as the spawning area [19]. Therefore, maintaining habitat connectivity between the TGR and the spawning grounds in the Jinsha River is very important for the survival and long-term persistence of LBG. Hence, fish passage facilities, especially upstream facilities, should be constructed in the dams downstream of LBG spawning grounds. 

## 5. Conclusions

This study of the diet shifts of a critically endangered fish species among three habitat types (SG, NRFA and TGRA) and between two ontogenetic stages (FMS and IS) showed that an obvious diet shift existed among three habitat types, but not between ontogenetic stages. Our results determined the food preference of LBG and confirmed the serious intraspecific competition between immature and fully matured individuals in a resource-limited area, both of which can guide future work conducted in captive breeding (aiming to restore the wild population of LBG via artificial release) of LBG. The results also showed that the formation of the Three Gorges Reservoir (TGR) in the upper Yangtze River altered the trophic structure, likely providing more high-quality food organisms. However, due to the cascade hydropower construction of the Jinsha River, it is necessary to maintain the habitat connectivity between the TGR and the spawning grounds in the Jinsha River for LBG survival and persistence over the long term [57]. In this context, the construction of fish passage facilities in the downstream LBG spawning grounds in the lower reaches of the Jinsha River should be implemented in the near future. In addition, due to significant differences in diet compositions among different critical habitats, a sufficiently broad view of trophic ecology, especially in regard to the food preference of a single fish species, made it clear that the effects of habitat types must be fully investigated. 

This study was in compliance with the International Union for Conservation of Nature (IUCN) Policy Statement on Research Involving Species at Risk of Extinction because the species *C. guichenoti* was not be listed as an endangered species during the sampling period (5 June to 16 June 2013), and this species was listed as critically endangered on the Red List of China’s Vertebrates in 2016.

## Figures and Tables

**Table 1:** Comparisons of the basic biological parameters among three habitat types in the upper Yangtze River.

Biological Parameter		SG		NRFA		TGRA	
		Immature	Fully Mature	Immature	Fully Mature	Immature	Fully Mature
Total length (mm)	Mean ± SD	233 ± 60 ^a^	368 ± 42 ^b^	289 ± 63	none	286 ± 47	none
	Range	95–343	304–409	131–396	none	194–346	none
Standard length (mm)	Mean ± SD	194 ± 52 ^a^	307 ± 35 ^b^	240 ± 53	none	238 ± 41	none
	Range	75–292	260–405	107–331	none	157–291	none
Body weight (g)	Mean ± SD	128.9 ± 92 ^a^	524.8 ± 236.7 ^b^	262.1 ± 175.4	none	233.2 ± 114.0	none
	Range	5.5–418.6	235.1–1359.9	19.4–866.4	none	52.7–411.7	none
Age (years)	Mean ± SD	1.79 ± 0.64 ^a^	2.46 ± 1.11 ^b^	2.47 ± 0.79	none	2.40 ± 0.72	none
	Range	1–3	3–6	1–4	none	1–3	none
N		43	25	55	0	52	0

Notes: SD, standard deviation; N, numbers of specimens. Different lowercase letters (^a,b^) indicate significant differences between immature individuals and fully mature individuals; SG, the spawning ground; NRFA, the natural riverine feeding and nursery area; TGRA, the Three Gorges Reservoir area; none, no samples were collected.

**Table 2:** Comparisons of the mean values of various physical and chemical parameters among the three habitat types (spawning ground (SG), natural riverine feeding and nursery area (NRFA) and Three Gorges Reservoir area (TGRA)).

Parameter	SG	NRFA	TGRA	*p*
Dissolved oxygen (DO, mg/L)	9.19 ± 0.20	7.64 ± 0.32	8.04 ± 0.51	0.013
pH	8.13 ± 0.08	8.38 ± 0.04	8.48 ± 0.02	0.001
Total phosphorus (TP, mg/L)	0.08 ± 0.02	0.13 ± 0.01	0.10 ± 0.01	0.018
Total nitrogen (TN, mg/L)	2.36 ± 0.91	2.32 ± 0.18	2.55 ± 0.27	0.933
Chemical oxygen demand (COD_Mn_, mg/L)	2.83 ± 1.33	2.68 ± 0.18	2.57 ± 0.23	0.946
Biochemical oxygen demand (BOD_5_, mg/L)	1.11 ± 0.56	1.95 ± 0.55	1.50 ± 0.70	0.436
Velocity of water flow (Velocity, m/s)	2.29 ± 0.11	1.87 ± 0.20	0.59 ± 0.13	<0.001
Water temperature (T, °C)	20.17 ± 0.62	22.60 ± 0.08	23.37 ± 0.62	0.002
CPB (%)	54.61 ± 8.56	34.90 ± 5.33	7.72 ± 2.56	<0.001
PPG (%)	28.46 ± 10.96	36.63 ±6.26	12.28 ± 6.25	0.002
SPS (%)	16.93 ± 4.35	28.48 ± 3.08	80.01 ± 6.81	<0.001

Notes: Results are from one-way ANOVA or Kruskal-Wallis tests; *p*, significance probability; CPB, cobble plus boulder; PPG, pebble plus gravel; SPS, sand plus silt; SG: spawning ground; NRFA, the natural riverine feeding and nursery area; TGRA, the Three Gorges Reservoir area.

**Table 3:** Mean δ^13^C and δ^15^N values (±SD) of four food sources from three habitat types in the upper Yangtze River.

Food Sources		δ^13^C				δ^15^N			
		SG	NRFA	TGRA	*p*	SG	NRFA	TGRA	*p*
Mollusks	Mean	−20.03	−23.41	−23.24	0.001	9.78	9.72	6.78	0.012
	SD	0.52	0.21	0.17		0.41	0.14	0.24	
Macrocrustaceans	Mean	−24.81	−24.06	−25.11	<0.001	10.42	6.24	5.21	0.007
	SD	0.33	0.48	0.47		0.37	0.32	0.32	
Aquatic insect larvae	Mean	−25.24	−24.08	−23.18	<0.001	8.78	7.12	6.12	<0.001
	SD	0.22	0.21	0.53		0.19	0.54	0.45	
POM	Mean	−24.78	−22.13	−21.42	0.002	6.79	4.78	3.11	<0.001
	SD	0.82	0.19	0.09		0.53	0.47	0.08	

Notes: Results are from one-way ANOVA or Kruskal-Wallis tests; *p*, significance probability; SD, standard deviation; N, numbers of samples. SG: spawning ground; NRFA, natural riverine feeding and nursery area; TGRA, Three Gorges Reservoir area; POM, particulate organic matter.

**Table 4:** Percentages of potential food contributions (50% quantiles, range = 95% Bayesian credible intervals) in three sampling sites for *Coreius guichenoti* estimated using MixSIAR.

Potential Food Sources	SG		NRFA		TGRA	
	50%	Range	50%	Range	50%	Range
Aquatic insect larvae	0.5	0.1–3.2	1.6	0.1–8.7	0.7	0.1–5.5
Macrocrustaceans	3.4	0.9–8.1	56.4	16.8–82.8	49.4	35.6–65.0
Mollusks	3.7	0.4–13.3	6.1	2.7–11.0	47.4	31.9–61.4
POM	91.0	81.2–97.3	34.4	8.9–73.9	1.4	0.5–3.4

Notes: SG: spawning ground; NRFA, the natural riverine feeding and nursery area; TGRA, the Three Gorges Reservoir area; POM, particulate organic matter.

**Table 5:** Percentages of potential food contributions (50% quantiles, range = 95% Bayesian credible intervals) in two ontogenetic stages for *Coreius guichenoti* estimated using MixSIAR.

Potential Food Sources	FMS		IS	
	50%	Range	50%	Range
Aquatic insect larvae	5.2	0.4–17.4	1.0	0.1–4.1
Macrocrustaceans	2.7	0.2–10.2	0.6	0.1–3.0
Mollusks	4.3	0.5-–13.1	3.0	0.1–13.4
POM	85.1	74.4–93.7	94.2	84.3–98.9

Notes: FMS, fully mature stage; IS, immature stage; POM, particulate organic matter.
